# lncRNAs: a new generation of targets and biomarkers in thyroid cancer

**DOI:** 10.1530/ETJ-25-0290

**Published:** 2026-01-19

**Authors:** Rufina Maturi, Matteo Esposito, Rob P Coppes, Gabriella De Vita

**Affiliations:** ^1^ Department of Molecular Medicine and Medical Biotechnology, University of Naples “Federico II”, Naples, Italy; ^2^ Department of Radiation Oncology, University Medical Center Groningen, University of Groningen, Groningen, The Netherlands; ^3^ Department of Biomedical Sciences, University Medical Center Groningen, University of Groningen, Groningen, The Netherlands

**Keywords:** lncRNA, thyroid cancer, therapeutic target, biomarker

## Abstract

Long non-coding RNAs (lncRNAs) are untranslated RNA molecules that regulate gene expression through diverse mechanisms, acting as scaffolds, guides, decoys, or signals. In thyroid cancer, the most prevalent endocrine malignancy, lncRNAs are increasingly recognized as key contributors to tumor development and progression. Elucidating these molecular mechanisms is essential for advancing diagnostic, prognostic, and therapeutic strategies. This review highlights major lncRNAs implicated in thyroid cancer, categorizing them as upregulated/oncogenes or downregulated/tumor suppressors and describing their mechanisms of action and interactions. lncRNAs are typically expressed at low levels and tightly regulated to preserve normal cell behavior. In thyroid cancer, they serve as crucial regulators of oncogenesis, frequently acting as competing endogenous RNAs that influence key signaling pathways. While most studies focus on miRNA sponging, other mechanisms are underexplored. Circulating lncRNAs offer potential for non-invasive diagnostics, and several lncRNAs show promise as therapeutic targets. Thus, continued research into the diverse functions of lncRNAs is vital to fully harness their clinical potential in thyroid cancer.

## Introduction

**Figure 1:**
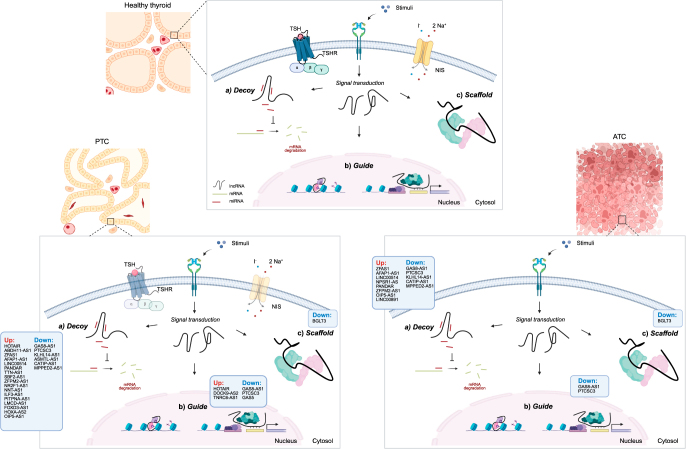
(Top) Schematic overview of the known modes of action of lncRNAs in healthy thyroid cells. (Bottom left) Summary of the up-to-date described lncRNAs dysregulated in PTC (divided into up- and down-regulated) and relative modes of action. (Bottom right) Summary of the up-to-date described lncRNAs dysregulated in ATC and PTC (divided into up- and down-regulated) and relative modes of action. lncRNAs reported in the figure include both the ones described in the main text and the ones described in the Supplementary section (see section on Supplementary materials given at the end of the article).

lncRNAs are non-translated transcripts longer than 200 bp (or 500 bp according to a recent consensus statement paper), encoded by intergenic regions or overlapping, partially or entirely, with protein-coding genes; however, despite the proximity, their transcriptional regulation often remains independent of nearby protein-coding genes (1). lncRNAs are involved in a wide range of physiological and pathological processes by regulating gene expression through *in cis* or *in trans* mechanisms: they can act at the epigenetic, transcriptional, post-transcriptional, translational, and post-translational levels. These molecules can be classified by their mode of action into the following:

- Decoys: sponge RNAs or miRNAs preventing their binding to targets.
- Guides: direct proteins to specific genome regions, through sequence complementarity, to regulate transcription or chromatin structure.
- Scaffolds: promote protein–protein, protein–DNA, or protein–RNA interactions through binding by base-pairing of their primary sequence and/or by three-dimensional structures (2) (Fig. 1).

Unequivocally, most of the lncRNAs have been identified through a precise and straightforward experimental scheme as miRNA sponges, while there is still limited information on lncRNAs acting as guides or scaffolds due to the lack of standardized experimental tools. This implies that the unexplored aspects of these molecules could conceal significant information that can be utilized to better address these neoplasms.

Due to their diverse roles, lncRNAs are crucial in numerous biological processes, and their dysregulation significantly contributes to disease pathogenesis. Many are associated with the onset and progression of cancer through altered expression and/or mutations in their genes, functioning as oncogenes or tumor suppressors.

Thyroid cancer (TC) is the most common endocrine tumor and the seventh most prevalent malignancy worldwide, with 821,214 cases diagnosed and 47,507 deaths annually (0.44% of all cancers) (WHO, Feb 2025). TC incidence has risen globally, although mortality has slightly declined. This observation led to the hypothesis that the higher incidence may be due to advancements in detection methodologies (3). Over 90% of diagnosed TCs are well-differentiated papillary, follicular, or oncocytic and generally have favorable outcomes (4). TC cells can accumulate genetic mutations over time, resulting in rarer and more aggressive subtypes, including poorly differentiated and anaplastic thyroid carcinomas (5). Depending on the histotype and aggressiveness of the tumor, therapeutic approaches can vary, from surgery to radiotherapy to targeted therapy. This underscores the importance of identifying molecular targets to enhance our ability to combat tumor cells and utilize them as prognostic markers. Indeed, expanding our understanding of tumor development is foundational for improving treatments and follow-up care. This review outlines current knowledge on lncRNAs in TC, distinguishing between upregulated (oncogenes) (Table 1) and downregulated (tumor suppressors) transcripts (Table 2), also highlighting their potential application as therapeutic, prognostic, or diagnostic factors.

**Table 1:** Summary of the main lncRNAs upregulated in thyroid cancer, including the mode of action, intermediate molecules, target dysregulated genes, and their potential application as diagnostic, prognostic, and/or therapeutic tools.

lncRNA/mode of action	Mediator	Target	Potential application
HOTAIR			
Sponge	miRNA-1	CCND-2	
Sponge	miRNA-17-5p	Unknown	Diagnostic
Sponge	miR-761	PPME1	Prognostic
Sponge	miR-488-5p	NUP205, Bcl2	Therapeutic
Guide	miR-181a	GATA6	
ABHD11-AS1			
Sponge	miR-1301-3p	STAT3	Diagnostic
Unknown	Unknown	EPS15L1	Prognostic
Sponge	miR-199a-5p	SLC1A5	Therapeutic
ZFAS1			
Sponge	miR-150-5p	Unknown	Diagnostic
Sponge	miR-590-3p	HMGA2	Prognostic
AFAP1-AS1			
Sponge	miR-155-5p	ETS1	Therapeutic
Sponge	miR-204-3p	DUSP4	Prognostic
LINC00514			
Sponge	miR-204-3p	CDC23	Therapeutic
DOCK9-AS2			
Guide	SP1	CTNNB1	Diagnostic
Sponge	miR-1972	CTNNB1	Therapeutic
NPSR1-AS1			
Scaffold	ELAVL1	NPSR1	Therapeutic
TNRC6-AS1			
Unknown	TNRC6	Iodine metabolism genes	Diagnostic
Guide	DNA methyltransferases	STK4	Therapeutic

**Table 2:** Summary of the main lncRNAs downregulated in thyroid cancer, including the mode of action, intermediate molecules, target dysregulated genes, and their potential application as diagnostic, prognostic, and/or therapeutic tools.

lncRNA/mode of action	Mediator	Target	Potential application
GAS8-AS1			Diagnostic
Sponge	miR-135b-5p	CCNG2	
Sponge	miR-187-3p	ATG5	
Sponge	miR-1343-3p	ATG7	
Guide	MLL1/WDR5	GAS8, AFAP1-AS1, UCA1	
PTCSC3			Diagnostic
Sponge	miR-574-5p	SCAI	
Unknown	Unknown	LRP6, Axin	
Unknown	Unknown	YAP/TAZ	
Guide/mRNA destabilization	Unknown (probably RBPs)	STAT3	
RMST			Diagnostic
Guide	SOX2	NANOG, OCT4, SOX2, SNAI1, SLUG, TWIST	
PAR5			Diagnostic
Scaffold	EZH2	EGFR, VEGF-A, CCNA1, CDH1	
KLHL14-AS1			
Sponge	miR-182-5p	BCL2, PAX8	Diagnostic
Sponge	miR-20a-5p	PAX8	Therapeutic
BGLT3			Diagnostic
Scaffold	OTUD3	PTEN	
Scaffold	BRCA1/BARD1	DDR	
Scaffold	PARP1	DDR	
ASMTL-AS1			Diagnostic
Sponge	miR-1228-3p	SOX17	
Sponge	miR-93-3p, miR-660	FOXO1	
CATIP-AS1			
Sponge	miR-515-5p	SMAD4	Diagnostic
MPPED2-AS1			
Decoy	DNMT1	MPPED2	Diagnostic
GAS5			
Sponge	miR-221-3p	CDK6	Diagnostic
Sponge	miR-196a-5p, miR-182-5p	FOXO1, FOXO3	Therapeutic

## Upregulated lncRNAs

### HOTAIR

Homeobox transcript antisense RNA (*HOTAIR*) is transcribed from the antisense strand of the HOXC cluster locus, serving as a scaffold for two key histone modification complexes. It binds to the polycomb repressive complex 2 (PRC2) to epigenetically repress transcription from the *HOXD* locus, and it interacts with the lysine-specific demethylase 1 (LSD1)-CoREST complex to remove histone markers linked to gene activation (6). Recently, *HOTAIR* has been implicated in TC with two independent studies highlighting its upregulation in TC samples (7, 8). Zhang *et al.* later showed *HOTAIR* is also present in the plasma of TC patients, and, through *in vitro* analyses, they demonstrated that it acts as an oncogene, controlling cancer proliferation and invasion, linking its expression to the progression and prognosis of thyroid carcinoma (7). Furthermore, *HOTAIR* overexpression in the lymph node metastasis of papillary thyroid cancer (PTC) suggests its role in epithelial-to-mesenchymal transition (EMT) *via* the Wnt/β-catenin pathway, by inducing *SNAIl* and *ZEB1*, which enhance migration. Silencing *HOTAIR* downregulates β-catenin and enhances Wnt inhibitor 1 (*WIF1*), reducing invasiveness (9). Furthermore, it sponges tumor suppressive miRNAs, such as miR-1, thus enhancing cyclin D2 (CCND-2) and contributing to tumor progression (8). Other target miRNAs are miR-17-5p and miR-761, enhancing cell viability, migration, and invasion (10). While miR-17-5p exact mechanism is still unknown, miR-761 binds protein phosphatase 2A (PP2A)-specific methyl esterase (PPME1). This protein, a molecular target in various tumors, is positively correlated with Erk signaling (11): *HOTAIR* upregulation limits miR-791 availability, reinforcing PPME1’s promotion of the oncogenic Erk pathway (12). In addition, *HOTAIR* baits miR-488-5p, boosting nucleoporin 205 (NUP205) and the apoptotic protein BCL-2, thereby aiding tumor growth (13). Under hypoxia, a crucial tumor promoting condition (14), *HOTAIR* recruits RELA, a transcription factor from the NFκB/RelA family, which in turn activates miR-181a. The latter is a component of cancer-secreted exosomes, recently identified as an oncomiR in PTC (15). Presumably, miR-181a guides hypoxia-induced angiogenesis by diminishing GATA6 levels in target cells (16). Overall, *HOTAIR*’s upregulation is tied to multiple oncogenic pathways, highlighting its potential as a diagnostic and therapeutic target in TC.

### ABHD11-AS1

Located on chromosome 7, *ABHD11-AS1* regulates cell proliferation, migration, and invasion in several cancers, and recently, it has been linked to TC. In PTC, its serum levels correlate with tumor size, stage, and lymph node metastasis, suggesting a link to poor prognosis (17). Moreover, *in vitro* and *in vivo ABHD11-AS1* knockdown inhibits cell proliferation and promotes apoptosis while also restraining tumor growth and metastasis. At the molecular level, the cancer-related protein STAT3 was found to be responsible for the transcriptional activation of *ABHD11-AS1* in PTC; conversely, the lncRNA sponges miR-1301-3p, which causes upregulation of *STAT3* mRNA levels. This mechanism defines a positive feedback loop involving *ABHD11-AS1* and STAT3 that activates the downstream tumor-promoting PI3K/AKT pathway, thereby elucidating the oncogenic function of this lncRNA (18). Furthermore, a recent study highlighted a link between *ABHD11-AS1* and EPS15L1, a substrate of EGFR tyrosine kinase activity involved in the regulation of cell proliferation, differentiation, growth, and survival. It was shown that overexpression of *ABHD11-AS1* enhances EGFR, STAT3, p-STAT3, and EPS15L1, although the specific miRNA regulating this entire process remains unidentified (17). Finally, it is known that *ABHD11-AS1* overexpression in advanced-stage PTC causes a miR-199a-5p-mediated increase in SLC1A5/ASCT2 levels. This molecule is a Na^+^-dependent neutral amino acid transporter that functions as an oncogene in many human cancers, primarily by transporting glutamine to support tumor growth (19). *SLC1A5* is expressed exclusively by BRAF p.V600E mutated tumor cells in the thyroid (20). These data indicate that *ABHD11-AS1* is a tumor promoter in TC, and it could be employed as a diagnostic and prognostic factor; moreover, its interplay with the PI3K/AKT pathway and glutamine transport paves the way for considering it as a therapeutic target.

### ZFAS1

*ZFAS1* (ZNFX1-antisense-RNA1) is transcribed antisense to the protein-coding *ZNFX1* gene and accommodates three small nucleolar RNAs. It has been depicted as tumor suppressor in some tumors, while it acts as an oncogene in others, such as colorectal and gastric tumors (21, 22). Recently, *ZFAS1* has been found overexpressed in human TC tissues, and its upregulation occurs from the early stages of neoplastic transformation, correlating with TNM stage, lymph node metastasis, and recurrence. In human anaplastic TC (ATC) cells, *ZFAS1* knockdown decreased proliferation and cell cycle arrest: bioinformatic analyses predicted that it may be part of a competing endogenous RNA (ceRNA) circuitry involving miR-150-5p and miR-590-3p, along with more than one hundred mRNAs associated with DNA replication, ribosome function, transcription, translation, ubiquitin-mediated proteolysis, and sister chromatid separation. Despite the need for further validations, these findings suggest that *ZFAS1* may serve as a promising biomarker and prognostic factor for TC (23). In PTC, it stimulates proliferation while inhibiting apoptosis, promoting tumor growth *in vivo*. Also here, it sponges the tumor suppressor miR-590-3p, thereby upregulating HMGA2, a transcriptional regulatory factor well-recognized as a biomarker for TC (24). *ZFAS1* is a direct p53 target: in its wild-type form, *TP53* suppresses *ZFAS1* via miR-135b-5p and miR-193a-3p, whereas mutated *TP53* fails to do so, contributing to oncogenesis (25). Doubtlessly, this lncRNA can be considered pivotal for TC development and evolution, although plenty of information still needs to be uncovered. Indeed, the *ZFAS1*/miR-590-3p/HMGA2 axis or the interaction with p53 is probably only a small piece in the complex mechanisms linking *ZFAS1* to TC.

### AFAP1-AS1 and LINC00514

Actin filament-associated protein 1-antisense RNA 1 (*AFAP1-AS1*) is a tumor promoter in many malignancies, such as esophageal adenocarcinoma and colorectal cancers (26, 27). Recent findings indicate that *AFAP1-AS1* is overexpressed in ATC, where it sponges miR-155-5p, derepressing ETS1, a transcription factor that regulates many cancer-related genes, including *RAS*, *MET*, and also ERK phosphorylation (28). In differentiated TC, *AFAP1-AS1* expression is also elevated, with lower levels of this lncRNA correlating to better survival rates, thus establishing a new prognostic marker. *In vitro* analyses of human differentiated TC cell lines revealed that *AFAP1-AS1* knockdown inhibited tumor growth, promoted apoptosis, and hindered migration through EMT, although no molecular mechanisms were described (29). A separate study showed that *AFAP1-AS1* overexpression in TC tissues sequesters miR-204-3p, which normally regulates dual specificity phosphatase 4 (DUSP4) mRNA levels, thereby leading to its enhancement (30). DUSP4, a member of the mitogen-activated protein kinase phosphatase 2 (MKP2) family, plays a crucial role in dephosphorylating and inactivating MAPKs, thereby fine-tuning proliferation (31). Moreover, in PTC, *DUSP4* overexpression correlates with BRAF p.V600E mutation, highlighting a potential biomarker for tumor aggressiveness associated with cancer progression and metastasis formation (32). Notably, miR-204-3p is part of another ceRNA network involving *LINC00514* and *CDC23*, which enhances the latter, thereby driving PTC progression (33).

### DOCK9-AS2

DOCK9 antisense RNA2 (*DOCK9-AS2*) is an exosomal lncRNA. Exosomes are small vesicles allowing cell to exchange molecules (34). Cancer cells exploit this communication mechanism to impact their environment; more specifically, cancer stem cells (CSCs) and non-CSCs use exosomes to affect each another, establishing a dynamic communication network (35). The interesting aspect of *DOCK9-AS2* is that, as an exosomal lncRNA, it can be delivered, exerting oncogenic functions in target cells. *DOCK9-AS2* is upregulated in PTC tissues and cell lines and has been detected in the exosomes of PTC patients. This is particularly relevant since detection of circulating lncRNAs, such as *HOTAIR* and *DOCK9-AS2*, opens avenues for non-invasive diagnostics and prognostic tools, considering also that in TC fine-needle aspiration cytology can sometimes yield inconclusive results. At the molecular level, it has been demonstrated that *DOCK9-AS2* enhances the Wnt/β-catenin pathway in two independent ways: in the nucleus, it recruits the tumor promoter transcriptional factor SP1 to promote *CTNNB1* transcription; in the cytoplasm, it sponges miR-1972 to stabilize *CTNNB1* mRNA (36, 37, 38). This evidence locates *DOCK9-AS2* as the first known lncRNA used as a signal by TC cells. Moreover, its implication in β-catenin regulation involves it in cancer progression and invasiveness.

### NPSR1-AS1

Neuropeptide S receptor 1 antisense RNA 1 (*NPSR1-AS1*), antisense to the *NPSR1* gene, is a rare case of scaffold lncRNA promoting proliferation, migration, and invasion in TC. Its silencing reduced these malignant behaviors and inhibited the EMT by increasing E-cadherin and decreasing N-cadherin and Vimentin levels (39). Mechanistically, *NPSR1-AS1* binds to the RNA-binding protein ELAVL1, stabilizing the mRNA of its nearby gene *NPSR1*. Elevated NPSR1 then activates the MAPK signaling pathway, contributing to TC progression. Thus, *NPSR1-AS1* enhances *NPSR1* expression and MAPK pathway activation via ELAVL1 interaction (39).

### TNRC6-AS1

*TNRC6-AS1* is a lncRNA transcribed from the reverse strand of trinucleotide repeat-containing 6C (*TNRC6C*). A recent study reported the oncogenic nature of *TNRC6-AS1* in PTC, linking its overexpression to increased proliferation, migration, and invasion rates. Interestingly, it is inversely correlated with the coding gene *TNRC6C*, which is repressed in thyroid tumor samples. Further analyses revealed that *TNRC6-AS1* affects the protein-coding gene, and they both influence iodine metabolism genes, such as *TSH-R*, *SLC5A5*, *TPO*, and *SLC26A4*, as the restoration of physiological expression levels of the two resulted in the rescue of the mentioned iodine-related genes. These findings may offer insights to improve response to radiotherapy, given that the iodine genes are essential for its outcomes and functioning (40). Furthermore, in PTC, *TNRC6-AS1* guides DNA methyltransferases on the CpG islands of the target gene serine/threonine-protein kinase 4 (*STK4*) promoter (41). Hypermethylation of CpG islands leads to silencing of the downstream gene, which can serve as a mechanism for the knockdown of tumor suppressor genes during cancer development and progression (42). STK4 downmodulation has been linked to the nuclear translocation of Yes-associated protein (YAP), promoting cell proliferation while inhibiting apoptosis and autophagy (41). YAP is a component of the Hippo pathway, a crucial signaling network for tissue development and regeneration, which is often dysregulated in cancer (43).

## Downregulated lncRNAs

### GAS8-AS1

*GAS8-AS1* is an antisense lncRNA transcribed on the opposite strand of the *GAS8* gene, with its transcription starting from a promoter region that remains to be fully characterized. This lncRNA was initially mapped on a region on chromosome 16q that is frequently deleted in prostate and breast cancers (44). The first evidence depicting *GAS8-AS1* as a tumor suppressor emerged from a whole-exome sequencing analysis conducted by Pan *et al.* (45) on a Chinese cohort of 91 paired PTC and normal tissues, in which this lncRNA was the second most frequently mutated gene with a significantly low expression in PTC samples (45). Upon the ectopic expression of wild-type *GAS8-AS1*, Pan and colleagues discovered that the proliferation of PTC and MTC cell lines was significantly hindered compared to controls, while the opposite occurred when the lncRNA was targeted by siRNAs (45). These findings were independently reproduced in hepatocellular carcinoma (46) and colorectal cancer (47) cell lines. Chen *et al.* found that *GAS8-AS1* indirectly regulates the stability of cyclin G2 mRNA (*CCNG2*) by sponging miR-135b-5p, a well-known oncomiR in many cancers, including PTC and MTC (48). This likely leads to an accumulation of cyclin G2 that delays cell cycle progression, which was measured in both cultured cells and tumor masses from nude mice grafted with *GAS8-AS1*-silenced cells (49). In addition, *GAS8-AS1* can promote uncontrolled proliferation and invasiveness in ATC (29, 50). Although the precise mechanism is unclear, both Zhao *et al. *(47) and Zha *et al. *(51) suggest that it can regulate transcription by recruiting epigenetic regulators. Indeed, this lncRNA can guide the epigenetic writer complex MLL1/WDR5 to trimethylate H3K4s on the *GAS8* gene promoter, facilitating the transcription of this tumor suppressor gene, which in turn regulates microtubule sliding during mitotic spindle assembly (46). Furthermore, *GAS8-AS1* also plays a role in the activation of autophagy, which is frequently hijacked by aggressive tumors as a form of metabolic adaptation to the new necessities of metastasizing cancer. Stress-related p38 activation of ATF2, a transcription factor commonly stimulated during autophagy, promotes the expression of *GAS8-AS1*, which prompts two effects. On the one hand, it hinders cell cycle by sponging miR-135b-5p, leading to the accumulation of cyclin G2. On the other hand, *GAS8-AS1* sponges miR-187-3p and miR-1343-3p, which downregulate ATG5 and ATG7, respectively, necessary for expanding the newly formed autophagosome (52). As Qin *et al.* demonstrated, the loss of *GAS8-AS1* in PTC removes one of the safeguard mechanisms against tumor progression while effectively rescuing its expression and sensitizes the tumor cells to autophagy, simultaneously decreasing the proliferation rate (53). Collectively, these findings suggest that alterations in *GAS8-AS1* function likely occur earlier in cancer onset, probably while the tumor remains well-differentiated rather than in aggressive tumors, as genetic hits to this tumor suppressor may help remove obstacles that advanced-stage tumors consider already surpassed.

### PTCSC3

*PTCSC3* is a tumor suppressor lncRNA, identified in thyroid after careful investigation of the surrounding locus in linkage with rs944289, a tag SNP on chromosome 14q13.3. Indeed, the systematic characterization of this lncRNA was conducted by Jendrzejewski *et al.*, who were the first to elucidate the biological significance of rs944289 as occurring in a proximal enhancer of a 1154 bp long non-coding RNA gene thereafter named papillary thyroid carcinoma susceptibility candidate 3 (54). Shortly after, Fan *et al.* studied the effects of reintroducing *PTCSC3* in thyroid carcinoma cell lines, where its expression is decreased. They reported the inhibition of cell growth upon *PTCSC3* recovery and identified oncomiR-574-5p as an interactor of this lncRNA (55). *PTCSC3* restoration negatively regulates the expression of the EF-hand calcium-binding protein S100A4, thereby limiting tumor invasiveness and reducing angiogenesis and extracellular matrix remodeling (56). *PTCSC3* also interferes with the dysregulated oncogenic Wnt/β-catenin pathway, which provides the survival advantage often observed in PTC (57). At least two research groups have linked tumor suppressor *PTCSC3* deregulation to the Wnt/β-catenin pathway, although parts of this complex regulatory process remain unknown. Xia *et al.* described the epistatic control of *PTCSC3* on the expression of Frizzled co-receptor LRP6 and scaffolder protein Axin in glioma cells (58). They showed that *PTCSC3* overexpression reduces LRP6 abundance while simultaneously exerting a significant positive regulation on both Axin transcript and protein. Consequently, the Wnt pathway becomes strongly desensitized by *PTCSC3* rescue, as the receptor is less prone to activation; meanwhile, the increased amount of Axin in the cytoplasm effectively targets β-catenin for degradation, reducing the expression of target genes *c-MYC* and *CCND1*. Axin also seizes YAP/TAZ in the cytoplasm, thereby lowering the expression of EMT genes *SNAI1* and *ZEB* while promoting the expression of *CDH1* in the process (58, 59). Later, Wang *et al.* demonstrated how *PTCSC3* stabilizes the transcript of suppressor of cancer cell invasion, *SCAI*, from miR-574-5p and explained how this pioneering transcription factor can recruit the SWI/SNF complex to suppress gene expression from both pathways (60, 61). The same conclusions, albeit in cervical carcinoma, have been independently reached by both Zhang *et al.* and Tong *et al.* (62, 63), further confirming this mechanism. Even more interesting is *PTCSC3* ability to directly modulate the half-life of STAT3 protein and its response to cytotoxic drugs. Indeed, Wang *et al.* discovered the presence of STAT3 protein in *PTCSC3* pulldown, and upon overexpression, the protein abundance of STAT3 decreased to 1/5 of baseline levels. This, in turn, triggered a downregulation of the downstream INO80 gene, increasing susceptibility to doxorubicin. ATC cells in which *PTCSC3* expression was restored expressed less P-glycoprotein (MDR1) and were more susceptible to doxorubicin treatment (64). Although the understanding of this lncRNA is far from complete, the evidence gathered thus far depicts *PTCSC3* as a fundamental ceRNA and epigenetic regulator that successfully restrains thyroid carcinoma from progressing into a more aggressive form.

### RMST

Rhabdomyosarcoma 2 associated transcript, or *RMST*, was first discovered in malignant soft tissue tumors with various degrees of musculoskeletal differentiation. Its sequence was cleverly reconstructed using library screening and RACE, after which a northern blot revealed a 1.25 kb long transcript mapping to chromosome 12q21 (65). The work of Uhde *et al.* and Chordoff *et al.* established *RMST* function in the brain, with the former group observing *RMST* expression extending from the dorsal midline to the most anterior tip of the telencephalon in the developing mouse brain, ultimately restricted to midline dopaminergic neurons in the adult mouse brain (66). Meanwhile, the latter group described the overall high conservation among orthologs from humans to frogs (67). Yakushina *et al.*, by examining eight microarray datasets and two validation RNA-seq datasets, demonstrated that *RMST* downregulation is a characteristic feature of ATC, linking dysregulated *RMST* to cancer progression and aggressiveness (68). De Martino *et al.* first systematically characterized *RMST* in thyroid carcinoma, showing its tumor-suppressive role in aggressive ATC and suggesting that its high expression in thyroid helps maintain thyroid cell differentiation (69). The authors showed that restoring *RMST* in ATC cells where it is absent leads to a sharp decrease in stemness markers, including NANOG, OCT4, and SOX2, resulting in a reduced proliferation capacity and increased apoptotic events, clearly suggested by the delayed decline in cell number and shrinkage of tumoral thyrospheres. Furthermore, they demonstrated how *RMST* can repress the mesenchymal markers SNAI1, SLUG, and TWIST1, significantly restraining the migration and invasion capacity of ATC cells. The evidence gathered aligns well with the proposed and verified mechanism of action of *RMST* in physiological development and pathological events, reaffirming its role as a key tumor suppressor in thyroid tissue (70, 71, 72).

### PAR5

*PAR5* is one of the transcripts characterized by Sutcliffe *et al.* originated from the Prader-Willi/Angelman region (PAR) on chromosome 15q11-13, whose alterations are responsible for the imprinting disorders Prader-Willi and Angelman syndromes. *PAR5* was initially identified through the northern blot as an ∼12 kb RNA enriched in skeletal muscle and brain (73). *PAR5* was first studied in high-grade glioma and glioblastoma by Zhang *et al.*, where its downregulation might help stratify patients with worse prognoses (74, 75). *PAR5* function is closely linked to that of EZH2. The interaction between lncRNAs and EZH2 is commonly observed, as this protein regularly binds *MALAT1* (76), *XIST* (77), and *HOTAIR* (6), although the dynamics by which *PAR5* modifies the PRC2 complex through its association with EZH2 depends on the circumstances. According to Wang *et al.*, *PAR5* directly interacts with EZH2 and SUZ12 in a manner that helps stabilize the PRC2 complex, resulting in a bona fide increased methylation activity on its targets, although they did not evaluate regulatory methylation on gene loci per se (78). This exploration, conducted in glioma-cultured cells, also revealed that restoring *PAR5* significantly reduced EGFR, VEGF-A, and cyclin A levels while decreasing AKT phosphorylation, highlighting this lncRNA tumor suppressor’s ability to direct methylation suppression on these oncogenes. The function observed by Wang and colleagues matches with the role of a *trans*-acting lncRNA already established in other instances (6, 77, 79), making *PAR5* appear mostly as a scaffolder/guide RNA of the PRC2 complex, although this interaction can be largely dependent on tissue-specific context. Indeed, on one pivotal work regarding the PAR5/EZH2 axis in thyroid oncology, Pellecchia *et al.* proposed a different mechanism for this lncRNA–protein complex. They demonstrated that *PAR5* downregulation is exclusive to ATC, and upon restoring *PAR5* in ATC cultured cells, it negatively impacts EZH2 stability through interaction while also significantly reducing PRC2-mediated H3K27 trimethylation around the *CDH1* promoter, consequently affecting proliferation and invasiveness (80).

### KLHL14-AS1

*KLHL14-AS1* is an intriguing thyroid-associated lncRNA, not only because it plays a tumor suppressor role but also for its specific expression in thyroid since the very early stages of thyrocyte specification during development. The first evidence of *Klhl14-AS1* emerged from its detection in a microarray of laser micro-dissected E10.5 developing mouse thyroid primordia by Fagman *et al.* (81). They discovered that *Klhl14-AS1* was the most enriched transcript (still under the RIKEN provisional name 4930426D05Rik) in the thyroid bud compared to the whole embryo, alongside *Bcl2* and *Pax8*. Afterward, Credendino *et al.* investigated the expression profile of *Klhl14-AS1* in adult mouse, showing that it is expressed in various tissues and confirming that mouse *Klhl14-AS1* is, in fact, highly conserved among mammals, to the extent that the entire locus and its vicinity are syntenic between humans and mice (82). Subsequent analysis by the same group revealed the role of mouse and human *KLHL14-AS1* as a tumor suppressor in TC (83). They demonstrated that downregulation of *KLHL14-AS1* increases cell viability and proliferation, and through RNA pulldown assays, they showed that it serves as a decoy for miR-182-5p and miR-20a-5p, which target the mRNA of two thyroid differentiation regulators, *PAX8* and *BCL2*. Interestingly, the interaction between *KLHL14-AS1* and these miRNAs is conserved across humans and rodents, emphasizing its significance in thyroid physiology and pathology.

### BGLT3

Beta globin locus transcript 3 (*BGLT3*) stands as an outsider in relation to the thyroid, as it subjected to the same transcriptional regulation as the β-globin-like locus on chromosome 11p15.4. In thyroid, *HBB*, the β-globin gene, is expressed in very low amounts and does not produce a significant quantity of protein, while *BGLT3* expression and function in erythroid cells is closely associated with the *BGLT3* and *HBG2* γ-globin genes, which are generally repressed in favor of the *HBB* gene upon the fetal-to-adult hemoglobin switch. Indeed, *HBG1/HBG2* gene expression is nearly zero, meaning that also *BGLT3* should go undetected. Intriguingly, *BGLT3* expression is regulated in normal thyroid by HIF1α, which is positively modulated by the PI3K and MAPK pathways (84). In the early phases of thyroid oncogenesis, BRAF p.V600E or RET/PTC oncogenes drive the expression of HIF1α (85), although the concomitant overexpression of *c-MYC*, belonging to the same signaling pathway, quickly overturns HIF1α regulation by suppressing *BGLT3* transcription at its locus (86). The current literature provides only one instance that explores *BGLT3* as a tumor suppressor in TC, namely the study by Zhao *et al.* In addition to demonstrating the repressive effect of c-MYC on the *BGLT3* promoter, they observed a pronounced attenuation of cellular proliferation, growth, and migration of PTC cells in which they restored *BGLT3* expression, attributing these effects to the increased stability of PTEN. They initially co-precipitated PTEN *via* RNA pulldown of *BGLT3*, proving the interaction between them. Subsequently, they reported PTEN reduced ubiquitylation, attributed to the interaction with this lncRNA–protein complex and OTUD3 deubiquitylase (87). However, presenting OTUD3 to PTEN is not the sole relevant function of BGLT3 in thyroid carcinogenesis as this lncRNA is also involved in DNA repair. Hu *et al.* found that silencing *BGLT3* caused severe chromosomal instability, with an increase in chromosome breaks, radial figures, and dicentric chromosomes (88). Interestingly, *BGLT3* is also a scaffold for the major protein involved in homologous recombination as the lncRNA binds to the C-terminal BCRT domain of BARD1, which then interacts with BRCA1, facilitating the recruitment of RAD51 to initiate strand recognition. Essentially, *BGLT3* acts as a tether that stabilizes the BRCA1/BARD1 complex to damaged DNA. In addition, *BGLT3* interacts with PARP1, directly bringing the lncRNA to sites of damage. Although this role of *BGLT3* has not been directly studied in TC, it holds significance because poorly differentiated thyroid carcinoma and ATC – the more aggressive and undifferentiated forms of TC – often exhibit complex genomes and chromosomal instability due to impaired DNA repair mechanisms (89).

### ASMTL-AS1

Acetylserotonin O-methyltransferase-like antisense RNA 1, or *ASMTL-AS1*, is a novel lncRNA that may play a role as either an oncogene or a tumor suppressor, depending on the cancer type. As a tumor suppressor, it has been shown to sponge miR-1228-3p, increase *SOX17* mRNAs, and suppress β-catenin expression in triple‐negative breast cancer (90). In PTC, *ASMTL-AS1* is frequently reported to be highly downregulated, which generally worsens the prognosis. Feng *et al.* found that *ASMTL-AS1* primarily resides in the cytoplasm of non-tumorigenic thyroid cells, where it can increase *FOXO1* expression by acting as a sponge for miR-93-3p and miR-660, thereby inhibiting their repressive activity on *FOXO1* mRNAs (91). The ectopic expression of *FOXO1* in PTC cells in which *ASMTL-AS1* is knocked down blocked the enhanced glycolysis induced by *ASMTL-AS1* loss, showing that FOXO1 mediates the inhibitory effects of the lncRNA on PTC cell proliferation. Interestingly, in PTC cells, *ASMTL-AS1* is transcriptionally activated by FOXO1 itself, creating a positive feedback regulatory loop that is crucial for suppressing PTC glycolysis and growth. Lower levels of *ASMTL-AS1* in PTC are associated with overexpression of miR-93-3p and miR-660, alongside reduced FOXO1 levels, leading to an increased glycolysis rate that provides sufficient energy for the uncontrolled growth of cancer cells.

### CATIP-AS1

lncRNA *CATIP-AS1*, or ciliogenesis associated TTC17 interacting protein antisense RNA 1, is significantly downregulated in both PTC and ATC, with its reduced expression correlating with a poor patient prognosis. According to Qi *et al.*, *CATIP-AS1* functions by directly sponging miR-515-5p in TC cells, thereby inhibiting its regulatory effect on the target mRNA *SMAD4*, a crucial mediator in the TGF-β signaling pathway (92). The upregulation of SMAD4 induced by overexpressed *CATIP-AS1* results in increased levels of epithelial markers, such as E-cadherin and ZO-1, while simultaneously reducing the expression of mesenchymal markers, such as N-cadherin and vimentin, thus inhibiting EMT. Consequently, the overexpression of *CATIP-AS1* has been shown to inhibit cell proliferation and migration while being associated with an increased rate of apoptosis, identifying *CATIP-AS1* not only as a tumor suppressor but also as a potential therapeutic target for managing aggressive TCs.

### MPPED2-AS1

Metallophosphoesterase domain-containing 2 antisense RNA 1, or *MPPED2-AS1* for short, previously known as RP5-1024C24.1, is a lncRNA located on chromosome 11p14.1 in an antisense orientation relative to the *MPPED2-AS1* gene, of which it is a natural antisense. *MPPED2* encodes a metallophosphodiesterase protein with tumor suppressor functions in various cancers, including cervical cancer, neuroblastoma, glioblastoma, and oral squamous cell carcinoma. Pellecchia *et al.* showed that *MPPED2-AS1* and *MPPED2* are both significantly downregulated in benign and malignant thyroid neoplasms, including well-differentiated and undifferentiated thyroid carcinomas, proving to be strongly positively correlated with each other (93). *MPPED2-AS1* positively regulates *MPPED2* expression by inhibiting DNA methyltransferase 1 (DNMT1), leading to decreased hypermethylation and inactivation of the *MPPED2* promoter. Overexpression of *MPPED2-AS1* in thyroid carcinoma cells has been shown to reduce cell proliferation and migration, partly by upregulating PTEN and decreasing AKT phosphorylation. Similar inhibitory effects on cell growth and migration were also observed with MPPED2 overexpression, suggesting that the tumor suppressive role of *MPPED2-AS1* is mediated through *MPPED2* induction. These findings indicate that *MPPED2-AS1*, along with MPPED2, is a critical tumor suppressor in thyroid carcinogenesis.

### GAS5

The growth arrest specific 5 gene, or *GAS5*, is transcribed into two major transcripts, named *GAS5a* and *GAS5b*, whose alternative splicing gives rise to two, among the at least 29 others, splice variants that regulate cell growth and proliferation in two opposite ways (94). Nevertheless, *GAS5* appears to be mostly downregulated (95), classifying it as a tumor suppressor in most tumors. A substantial amount of literature on *GAS5* demonstrates that it primarily acts as a negative regulator of the PI3K/AKT/mTOR pathway, thereby limiting the ability of the cell to proliferate (96). This is appealing to the TC discourse, as PI3K/AKT/mTOR is greatly active in RAS-driven follicular TC (FTC) and ATC. Indeed, a prospective study by Guo *et al.* found that GAS5 was significantly downregulated in TC, with no difference between histological types, compared to benign thyroid tissues (97). Moreover, lower *GAS5* expression correlates not only with poorer prognosis but also with an increased expression of *CDK6*, which is particularly overexpressed in FTC and can indirectly reinforce PI3K/AKT signaling. Indeed, it was Liu and colleagues who explained this peculiar behavior by showing how *GAS5*, acting as a sponge for miR-221-3p, preserves the levels of *CDKN2B* and its encoded protein, p15INK4b, which directly inhibits CDK6 by preventing its binding with D-type cyclins, thus hampering cell cycle progression (98). Moreover, GAS5 can exert indirect control over PI3K/AKT signaling by sponging miR-196a-5p and miR-182-5p, effectively to sustain the abundance of FOXO1 and FOXO3, two transcription factors that act redundantly and synergistically to arrest cell growth (96). Finally, *GAS5* influence over PI3K/AKT culminates in preserving the level of PTEN, a tumor suppressor itself that switches off the PI3K downstream signal and restricts cell proliferation (99). Recently, a preliminary study on PTC cell lines confirmed that ectopic expression of *GAS5* can positively regulate genes usually induced by IFN-α/IFN-β response, as IFI44, which is part of the negative feedback loop that interferes with JAK/STAT pro-survival signaling (100). Considering its intensive control over PI3K/AKT/mTOR and JAK/STAT pathways, *GAS5* is not only a good example of tumor suppressor lncRNA in thyroid but also a candidate for future RNA-based drug to take advantage of.

## Clinical translation of lncRNAs in thyroid oncology

Treatments and diagnostic procedures based on noncoding RNAs have been gathering the attention of researchers for nearly three decades, resulting in observational and interventional cancer clinical trials alike increasing in number (101). To date, there are no open or completed interventional clinical trials based on noncoding RNAs conducted for TC. This is partly due to the fact that the management of most TCs, especially differentiated thyroid carcinomas, has the highest efficacy among solid tumors (SEER, 2025).

Nonetheless, aggressive and treatment-refractory subtypes, such as radioiodine-refractory and anaplastic thyroid carcinomas, could benefit from novel molecular targets. As highlighted in this review, restoration of physiological lncRNA expression can mitigate tumor aggressiveness by reducing proliferation and invasiveness or promoting apoptosis. One potential strategy involves combining the restoration of basal levels of lncRNAs implicated in cancer cell dedifferentiation (e.g. *TNRC6-AS1* and *Klhl14-AS*) (40, 83) with radioactive iodine (^131^I) therapy. In this setting, the redifferentiation of cancer cells in both primary tumors and metastases could restore ^131^I uptake and treatment efficacy (102).

Another emerging approach could involve the rescue of lncRNAs regulating the production of proteins responsible for drug resistance and the drug itself, e.g. the case of *PTCSC3* and *MDR1* with doxorubicin susceptibility (64).

Current TC diagnosis and classification rely on established clinical workflows combining physical examination, serum TSH measurement, ultrasonographic risk stratification, and fine-needle aspiration biopsy (103). Nevertheless, the detectability of certain lncRNAs in patient serum (e.g. *HOTAIR* and *DOCK9-AS2*) suggests their potential as non-invasive biomarkers for post-treatment monitoring and disease surveillance (7, 36).

## Conclusion and future perspectives

In this review, we outline lncRNAs whose roles in TC have been at least partially elucidated, highlighting the various pathological contexts in which they are involved. Indeed, lncRNAs are associated with many modes of action; however, a review of the available literature reveals that over 90% of characterized tumor-linked lncRNAs function as sponges. As reported in this review, numerous studies have demonstrated that the upregulation of a lncRNA is crucial for generating downstream imbalances in various targets, each acting as bait for one or more miRNAs that subsequently dysregulate additional mRNAs. This suggests that each sponge lncRNA may have a multimeric effect. Indeed, the complex network of intracellular RNAs complicates the understanding of independent interactions and characterizes the intricate molecular mechanisms underlying tumorigenesis over time. For instance, *HOTAIR* is a tumor promoter in TC, and independent studies have defined its involvement in malignancy, culminating in an understanding of the aberrant effects of its overexpression, which occur through sequestering miRNA-1, miRNA-17-5p, miR-761, and miR-488-5p, consequently impacting the post-transcriptional miRNA-driven regulation of many other genes (8). From another perspective, there is still a lack of information in the literature about lncRNAs functioning as scaffolds or guides in TC. Notably, it has been reported that *TNRC6-AS1* acts as a tumor promoter by guiding DNA methyltransferase to the CpG island of the *STK4* promoter (41), while *DOCK9-AS2* directs the cancer-associated transcription factor SP1 to promote *CTNNB1* transcription (36). However, the roles of these specific types of lncRNAs in TC remain largely unknown, leaving a substantial gap in our understanding.

This review aims to highlight how lncRNAs are involved in every cellular process and, therefore, contribute heterogeneously to many hallmarks of cancer cells. Nonetheless, almost all the knowledge we have up to date remains solely related to the sponging mechanism, indicating that a significant number of processes continue to be overlooked.

Considering the promising results obtained with the manipulation of many lncRNAs on the aggressiveness of cancer cells, and also the opportunity to exploit some of them as biomarkers, it is undeniable that deepening this field may represent an effective new approach to the most common endocrine malignancy.

## Supplementary materials



## Declaration of interest

The authors declare that there is no conflict of interest that could be perceived as prejudicing the impartiality of the work reported.

## Funding

ME research fellowship was funded by the project T3-AN-09 by the Italian Ministry of Health. GDV, RPC, and RM received no funding to declare.

## Author contribution statement

RM: conceptualization (supporting). Writing – original draft (lead), and writing – review and editing (lead). ME: writing – original draft (supporting), and writing – review and editing (supporting). RPC: conceptualization (supporting), writing – review and editing (equal). GDV: conceptualization (lead), writing – review and editing (equal). All authors approved the final version of the manuscript.
