# Supplementary material for: lncRNAs: a new generation of targets and biomarkers in thyroid cancer
Source: Eur Thyroid J. 2026 Jan 19;15(1):ETJ250290. doi: 10.1530/ETJ-25-0290 (PMC12820940; doi:10.1530/ETJ-25-0290)
Supplement: Supplementary file 1 [file supplementary_materials.pdf]

Supplementary Table 1. Resume of other lncRNAs upregulated in thyroid cancer, including the mode of action, intermediate molecules and target dysregulated genes.

**lncRNA**	**Mode of action**	**Mediator**	**Target mRNA**
COMET	Unknown	Unknown	Unknown
PANDAR	Sponge	miR-637	KLK4
TTN-AS1	Sponge	miR-153-3p	ZNRF2
SBF2-AS1	Sponge	miR-431-5p	CDK14
ZFPM2-AS1	Sponge	miR-515-5p	TUSC3
NR2F1-AS1	Sponge	miR-423-5p	SOX12
NNT-AS1	Sponge	miR-199a-5p	unknown
ILF3-AS1	Sponge	miR-4306	PLAGL2
PITPNA-AS1	Sponge	miR-129-5p	UNC5B
LMCD-AS1	Sponge	miR-1287-5p	GLI2
FOXD3-AS1	Sponge	miR-296-5	unknown
HOXA-AS2	Sponge	miR-520c-5p	S100A4
HOXD-AS1	Unknown	Unknown	Unknown
LRRC52AS1	Unknown	Unknown	Unknown
OIP5-AS1	Sponge	miR-455-3p	MFAP2
LINC00891	Unknown	Unknown	EZH2<br>SMAD2/3
LINC00887	Unknown	Unknown	PD-L1
